# Maturation of the GABAergic Transmission in Normal and Pathologic Motoneurons

**DOI:** 10.1155/2011/905624

**Published:** 2011-07-20

**Authors:** Anne-Emilie Allain, Hervé Le Corronc, Alain Delpy, William Cazenave, Pierre Meyrand, Pascal Legendre, Pascal Branchereau

**Affiliations:** ^1^Institut de Neurosciences Cognitives et Intégratives d'Aquitaine (INCIA), Université de Bordeaux et CNRS—UMR 5287, avenue des Facultés, 4ème étage Est, 33405 Talence, France; ^2^INSERM U 952, CNRS UMR 7224, Université Pierre et Marie Curie, Bâtiment B, étage 2, boite postale 37, 7 quai Saint Bernard, 75005 Paris, France; ^3^Institut des Maladies Neurodégénératives (IMN), Université de Bordeaux et CNRS—UMR 5293, avenue des Facultés, 4ème étage Est, 33405 Talence, France

## Abstract

**γ**-aminobutyric acid (GABA) acting on Cl^−^-permeable ionotropic type A (GABA_A_) receptors (GABA_A_R) is the major inhibitory neurotransmitter in the adult central nervous system of vertebrates. In immature brain structures, GABA exerts depolarizing effects mostly contributing to the expression of spontaneous activities that are instructive for the construction of neural networks but GABA also acts as a potent trophic factor. In the present paper, we concentrate on brainstem and spinal motoneurons that are largely targeted by GABAergic interneurons, and we bring together data on the switch from excitatory to inhibitory effects of GABA, on the maturation of the GABAergic system and GABA_A_R subunits. We finally discuss the role of GABA and its GABA_A_R in immature hypoglossal motoneurons of the spastic (SPA) mouse, a model of human hyperekplexic syndrome.

## 1. Introduction

*γ*-aminobutyric acid (GABA) is, with glycine, the major inhibitory neurotransmitter in the adult central nervous system (CNS) of vertebrates. GABA acts on Cl^−^-permeable ionotropic   bicuculline-sensitive type A (GABA_A_) receptors (GABA_A_R) and metabotropic baclofen-sensitive GABA_B_R, these latter being coupled through G-proteins to K^+^ and Ca^2+^ channels in neuronal membranes. More recently, it has been shown that GABA also activates Cl^−^-permeable bicuculline- and baclofen-insensitive GABA_C_R, this receptor subtype being largely expressed in the retina and at lower level in other CNS area [1]. If all GABA receptors are present on the cell membrane, the common view is that GABA_B_R are presynaptically located, whereas GABA_A_R and GABA_C_R are postsynaptically. However, all GABA receptors seem to be located pre- and/or post-synaptically [2–5].

GABA is synthesized from the amino acid glutamate by the enzyme glutamic acid decarboxylase (GAD), this latter being present as two isoforms with different molecular weights of 65-kDa and 67-kDa [6]. The two GAD isoforms are product of two different genes. GAD65 gene (GAD2) is located on chromosome 10 (10p11.23) in human and on chromosome 2 (2 9.0 cM) in mouse, while GAD67 gene (GAD1) is located on chromosome 2 (2q31) in human and in chromosome 2 (2 43.0 cM) in mouse [7, 8]. In addition, during mouse and rat embryonic development, two alternatively splices forms are also synthesized from the GAD67 gene: the truncated 25-kDA leader (GAD25) and the enzymatically active protein GAD44 (for review, see [9]). GAD25 is a protein without GAD enzymatic activity. GAD25 and GAD44 are expressed during the development of the CNS, they are more abundant in proliferating progenitors [9–11], and they are downregulated during neuronal differentiation concomitant with an upregulation of GAD67 expression [12–14]. The 67-kDa GAD form is diffusely distributed in the cytoplasm of the cells, while the 65-kDa GAD form is mainly found attached to synaptic vesicles [15].

During CNS development, GABA exhibits a large panel of activity ranging from the control of cell proliferation to the formation of synapses (for review, see [16–19]). In immature brain structures, most studies described GABA as operating through GABA_A_R subclass [18, 20], and it was first proposed that the other GABAR subclasses were not functional at early stage of life [21]. However, this hypothesis was invalidated by the observation of a pre- and post-synaptic GABA_B_R expression in the embryonic rat neocortex [22] and the modulation of cortical neuronal migration by GABA_B_R activation [23–25]. GABA_B_R activation triggers BDNF release and promotes the maturation of GABAergic synapses [26]. Finally, it has been shown that GABA can control the locomotor network in the rat neonatal spinal cord by acting on presynaptic GABA_B_R as well as on postsynaptic GABA_A_R [27]. In the brainstem, it has been recently shown that the interaural time difference detection circuit is differentially controlled by GABA_B_R during the second post-natal week [28]. An endogenous modulation of respiratory rhythm by GABA_B_R that increases after birth has also been reported [29]. Finally, functional GABA_C_R were detected in the spinal motoneurons (MNs) around birth, but a little is known about the function of these receptors in the immature spinal cord [1].

GABA_A_R-related effects on immature neuronal cells are opposed to those observed on mature neurons in the sense that GABA exerts depolarizing effects during development, while it induces hyperpolarizing effects in most mature CNS regions [30]. Such depolarizing GABA-mediated effects, coupled with conventional excitatory effect of glutamate and other classical neurotransmitters such as acetylcholine, lead to Ca^2+^ influx and generate spontaneous electrical activities that are the features of almost all immature structures of the CNS [31, 32]. Numerous studies have demonstrated the permissive role of depolarizing GABA in the maturation of neurite outgrowth [33], in promoting both excitatory and inhibitory synaptogenesis [34] and in controlling its switch from depolarizing to hyperpolarizing [35, 36].

Brainstem and spinal motoneurons that are largely targeted by GABAergic interneurons require an appropriate maturation of their GABA receptors and GABA innervations. In the present paper, we will describe the ontogeny of the GABAergic system in spinal MNs in parallel to the establishment of an inhibitory transmission, and then we will present data about the maturation of GABA receptors in hypoglossal motoneurons (HMs, motoneurons innervating the tongue) of the spastic (SPA) mouse, a model of human hyperekplexic syndrome in which the impaired glycinergic neurotransmission [37] may be compensated, in certain strain lines, by an increased aggregation of GABA_A_R [38, 39]. The hyperekplexic syndrome, as well as the amyotrophic lateral sclerosis (ALS) pathology, highlights the plasticity of the GABAergic system that may temporally compensate genetic alteration of other inhibitory systems [40, 41].

## 2. Maturation of Chloride-Mediated Inhibition in MNs

GABA, when binding to GABA_A_R, exerts effects that are mainly dependent upon the chloride equilibrium potential (*E*_Cl_). In mature neurons, the intracellular Cl^−^ concentration [Cl^−^]_i_ is lower than extracellular Cl^−^ concentration [Cl^−^]_o_ and the activation of the chloride permeable channels by GABA induces a chloride influx. However, in immature neurons that express a higher [Cl^−^]_i_  compared to [Cl^−^]_o_, GABA acts as an excitatory neurotransmitter. Hence, during CNS development, a switch from excitatory to inhibitory effects of GABA occurs. In the mouse pre-Bötzinger complex (PBC), a brainstem respiratory structure that drives the rhythmic activity of the hypoglossal motoneurons, gramicidin perforated patch-clamp recordings that preserve the physiological [Cl^−^]_i_  indicate that the reversal potential of GABA_A_R-mediated current (EGABA_A_R that corresponds to *E*_Cl_) switches from depolarizing to hyperpolarizing within the first postnatal (P) week (EGABA_A_R drops from −44 mV at P2 to −71 mV at P4) [42]. Because the resting membrane potential (rmp) for all PBC neurons was −56 mV, a switch from excitatory to inhibitory effects of GABA is evidenced between P2 and P4. Results obtained from gramicidin perforate-patched HMs are in good agreement with those collected in PBC neurons, because *E*_Cl_ is measured as being −37 mV in neonates HMs (P2) and −73 mV in juveniles HMs (P15), but the exact time of the switch remains undetermined between P2 and P15 (rmp of HMs is −70 mV) [43]. However, two other studies [44, 45] reported that by birth, GABA induces a hyperpolarization of the membrane potential in respiratory medullary neurons and a suppression of respiratory frequency. These studies, which are based on gramicidin perforated-patch clamp recordings, rather indicate that the transition from excitatory to inhibitory effects occurs at approximately E19 but not during post-natal stages in respiratory networks. From a technical point of view, measures of the GABA_A_R-related driving force may be considered with caution because invasive recordings (including perforated patch-clamp) combined with large input resistances of immature neurons may lead to inexact resting membrane potential values, true resting membrane potential values being more hyperpolarized (see [46]).

**Figure 1:**
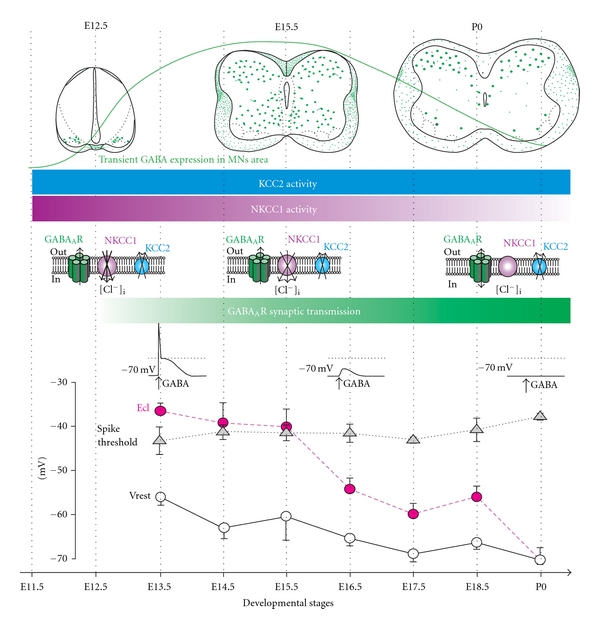
Development of the GABA_A_R-mediated inhibitory transmission in mouse lumbar spinal MNs. From top to bottom: schematic drawings (frontal views) depict the transient expression of GABA in spinal ventral interneurons (in green), while horizontal bars indicate the permanent KCC2 (in blue) and transient NKCC1 activity (in violet). The color intensity encodes the level of activity. NKCC1 inactivation combined to KCC2 activity leads to a significant decrease in [Cl^−^]_i_ and a disappearance of GABA_A_R-mediated excitatory effects. In parallel to the maturation of the chloride cotransporters KCC2 and NKCC1, the spinal cord starts to convey first synaptic activity at E12.5 that is GABAergic (green horizontal bar). Bottom: maturation of the chloride equilibrium potential (*E*_Cl_), spike threshold and resting membrane potential (Vrest) across the embryonic stages of developmental. Note the drop of *E*_Cl_ at E16.5 that accounts for the “shunting” GABA_A_R-mediated effect (modified from [47, 56, 66]).

When does the switch from excitatory to inhibitory effects of GABA occur in spinal MNs? We have showed that there is a shift of EGABA_A_R toward negative values during the embryonic development of mouse lumbar spinal MNs [47]. Our data demonstrated that until E15.5, *E*_Cl_ is above the spike threshold, whereas after E16.5, it drops significantly below spike threshold. During the course of the embryonic development, rmp of mouse spinal MNs remains below the *E*_Cl_. However, if GABA_A_R activation may trigger the firing of MNs until E15.5, after this embryonic developmental stage, such activation, although producing a depolarization, fails to trigger action potentials [47] (Figure 1). Our results indicate that GABA likely exerts a shunting action on mouse spinal MNs after E15.5, as demonstrated in the neonate rat spinal cord [48] and also described in current-clamp experiments by Hubner and collaborators in E18.5 mouse spinal MNs [49]. This shunting depolarizing GABA effect likely persists during postnatal stages even though our experimental measurements indicate that *E*_Cl_ reaches MNs rmp at P0 [47]. A recent study based on conventional intracellular recordings clearly demonstrated that the shift from excitatory to inhibitory IPSPs occurs at P4-5 in rat spinal MNs [50]. This was in agreement with intracellular recordings performed by Wu and collaborators showing much smaller (but still) depolarizing effects of GABA at P0 compared to E16–E18 in rat spinal MNs [51]. Another study, based on gramicidin perforated patch-clamp recordings, indicates that EGABA_A_R shifts between P5 and P10 in mouse spinal MNs, that is, at a later developmental stage compared to the rat [52]. Hence, further experiments would be needed to precisely determine whether the switch from excitatory to inhibitory effects of GABA really occurs in mouse spinal MNs, and it would be interesting to determine whether an oxytocin-driven transient loss of chloride occurs at birth in spinal MNs as described in hippocampal neurons [53].

## 3. Transient Expression of GABA in Motoneuronal Region during the Embryonic Life

Analyzing the maturation of GABA effects in MNs implies that an endogenous GABAergic innervation is present. GABA effects are indeed often tested using local application of exogenous GABA or GABA_A_R agonist (i.e., muscimol or isoguvacine) [42, 47]. It is thus essential to examine the ontogeny of GABA and GABA receptors. The detailed mapping of the GABAergic system has been extensively described in the adult brainstem by *in situ* hybridization, immunohistochemistry using antibodies directed against GABA or the GAD protein, specifically the 67-kDa isoform (GAD67) [54] or by taking advantage of the GAD67-GFP knock-in mouse [55]. However, to our knowledge, the ontogeny of the GABAergic innervation of brainstem MNs has not been precisely mapped.

We have described the process of embryonic maturation of GABA immunostaining in the mouse spinal cord [56]. Our study indicated that GABA-ir somata are first detected at embryonic day 11.5 (E11.5), exclusively at brachial level, in the ventral horn. By E13.5, the number of GABAergic neurons sharply increases throughout the extent of the ventral horn both at brachial and lumbar level. At E15.5, stained perikarya decrease in number in the ventral gray matter, while GABA-ir fibers are detected contacting MNs. Such a transient expression of GABA immunoreactivity in the spinal ventral horn was also described in the developing rat [57, 58] and chick [59].

## 4. GABAergic Synaptic Activity: A Predominant Neurotransmission in MNs at Early Developmental Stages

From a functional point of view, GABA effects differ according to the developmental stage. At early stages, excitatory GABA effects contribute, with cholinergic inputs, to the genesis of spontaneous network activity in the chick [60], mouse [61, 62] and rat [63, 64] spinal cord. At these early stages, MNs are still growing to their peripheral targets and the GABA-mediated spontaneous activity is required for correct motor axon guidance [65]. We have recently showed that first synaptic activity occurs at E12.5 in mouse spinal MNs [66] when the GABAergic phenotype starts to be largely expressed by interneurons located in the ventral gray matter [56]. GABAergic synaptic activity then increases in frequency and coexists with a glycinergic synaptic transmission [66]. In most immature CNS regions, GABA signaling is established before glutamatergic transmission, suggesting that GABA is the principal excitatory transmitter during early development [30]. In the spinal cord, pharmacological approaches performed while recording spontaneous activity showed as well that GABA generates, with acetylcholine [67], the earliest spontaneous motor activity and then glutamate interfere [64]. Our analysis also revealed that the glutamatergic synaptic transmission mainly develops in the embryonic spinal cord after the GABAergic one at around E14.5 (personal observation). Hence, GABA appears as a sort of automated expressed first ubiquitous signal, and then and only then does the adult behavior resumes. Interestingly, it has been shown that the glutamatergic transmission regulates the strength of GABAergic synapses [68].

If the synaptic transmission develops during the embryonic life in spinal MNs, it maturates during postnatal stages and a developmental shift from primarily long-duration GABAergic mIPSCs to short-duration glycinergic mIPSCs occurs after birth in rat MNs [69]. 

At E15.5 in the rat, commissural GABAergic connections mediate synchronous excitatory action on rhythm-generating networks in the ventral spinal cord, while at E18.5, these GABAergic commissural connections are responsible for reciprocal inhibition during left and right alternation [70]. Interestingly, at E20.5 in rat embryo, these inhibitory commissural inputs become mediated by glycine and not anymore by GABA [70]. These results that take over the primordial role of GABA_A_R for ensuring spontaneous activity and then reciprocal inhibition between left and right sides of the ventral spinal cord may explain why such a huge expression of GABA is detected in ventral spinal networks at E15.5, in the mouse [56]. At postnatal developmental stages, when commissural connections are mostly mediated by glycine [71–74], GABAergic inhibition has been shown to regulate the onset and duration of neurochemically induced locomotor activity [75].

## 5. Ontogeny of KCC2 and NKCC1 Immunoreactivity

The switch from excitatory to inhibitory GABA_A_R-related effects is closely related to the lowering of [Cl^−^]_i_  during the course of the development. This latter mainly relies on the differential ontogenic expression of the Na^+^/K^+^/2Cl^−^ cotransporter isoform 1 (NKCC1), which uptakes chloride ions [76–78], and the neuronal K^+^/Cl^−^cotransporter type 2 (KCC2) [79], which extrudes chloride ions [49, 80]. However, other exchangers can control the chloride gradient as the anion (Cl^−^–HCO_3_^−^) exchangers, either Na^+^- independent (AE) or Na^+^-driven (NDCBE also called NDAE) [81] (NCBE) [82]. AE mediates influx of Cl^−^ while exporting HCO_3_^−^, these exchanges being triggered by intracellular alkalinisation. NDCBE, known as an acid extruder (extrudes H^+^), moves Cl^−^ out in exchange of HCO_3_^−^, driven by the Na^+^ gradient [83, 84]. NCBE also lowers [Cl^−^]_i_  (and [H^+^]_i_) while importing Na^+^ and HCO_3_^−^ [82, 85].

It is generally accepted that early in development, NKCC1 is predominant and, therefore, maintains a high [Cl^−^]_i_, while at later stages, NKCC1 vanishes, and KCC2 develops, lowering intracellular chloride levels [86–88]. In spinal cord MNs, it was shown that the expression of KCC2 transcripts parallels neuronal differentiation during the embryonic life and preceded the decline of the GABA_A_R reversal potential (EGABA_A_R) [52]. Thus, the relationship between KCC2, NKCC1, and EGABA_A_R during the course of the embryonic development remained an open question. We addressed this question in a previous study [47] and found that KCC2 immunoreactivity (KCC2-ir) can be detected in MNs area as early as E11.5, confirming the Stein's study [52], when NKCC1 is also largely expressed. At E14.5, KCC2 is largely present in the ventral gray matter and at later stages this protein keeps stable. At E11.5, a dense NKCC1 labelling is detected throughout the ventral grey matter. Thus, our data indicated that the main drop of *E*_Cl_ occurring at E16.5 is likely dependant on a reduction of the NKCC1 efficacy rather than a later expression of KCC2. In the rat, Stil and co-workers investigated the expression of KCC2 and NKCC1 in the ventral horn of the spinal cord from E17 to P20 and found that the expression of KCC2 increases significantly, while the expression of NKCC1 decreases during postnatal life when the shift from depolarizing to hyperpolarizing IPSPs occurs (at P4-P5) [50].

It must be mentioned that analyzing the shift from depolarizing to hyperpolarizing effects of GABA in spinal MNs by taking into account only KCC2 and NKCC1 may be simplistic, because the anion exchangers AE has been clearly demonstrated as accumulating chloride in immature chick MNs [89]. Hence, the expression of inhibitory GABA effects likely also relies on the reduction of AE in addition to NKCC1. Also, NCBE that is expressed as early as E14.5 in the mouse SC [90] may play an important role in lowering [Cl^−^]_i_.

On the whole, even though likely oversimplified, Figure 1, that is based on our data, illustrates the ontogeny of the GABAergic inhibitory synaptic transmission in parallel to the activity of the two main cotransporters KCC2 and NKCC1. It must be noted that the transient maximum expression of GABA in ventral motor network precedes the drop of *E*_Cl_.

## 6. Ontogenic Changes of the GABAergic Receptors in MNs

GABA_A_R and GABA_C_R as glycine, nicotinic acetylcholine, and 5-hydroxytryptamine type 3 receptors belong to the cystein-loop receptor family. They are both pentameric assemblies of subunits, each subunits being characterized by extracellular N and C terminals and by four transmembrane domains (TM1–TM4), the domain TM2 forming the anionic channel pore [91]. GABA_A_Rs are composed of a large variety of different subunits, sixteen GABA_A_Rs subunits being cloned so far (*α*1–6, *β*1–3, *γ*1–3, *δ*, *ε*, *θ*, and *π*) and three (*ρ*1–3) for GABA_C_R [92, 93]. The number of GABA_A_R subunits is also theoretically increased by alternative splicing but only a dozen of subunit combinations have been detected so far [93]. The agonist binding site is carried mainly by *α* subunits, while *γ* subunits are responsible for linking GABA_A_Rs to the postsynaptic cytoskeleton. The most abundantly expressed GABA_A_R in the adult CNS has a stoichiometry of 2*α*, 2*β*, and 1*γ*2 subunit. In addition GABA_A_R subunit combination varies according to the synaptic and extrasynaptic location of this receptor. For example, GABA_A_Rs containing the *δ* subunit or the *α*5 subunit cannot accumulate at postsynaptic site, likely because they cannot anchor to postsynaptic scaffold protein complex [93–95]. Remarkably, the extrasynaptic GABA_A_Rs containing the *δ* subunit (*αβδ* GABA_A_R) have a higher apparent affinity for GABA and desensitize more slowly and less extensively than postsynaptic GABA_A_Rs containing the *β* and/or the *γ* subunits [96], while GABA_A_Rs containing the *α*5 subunit display a reduction in their desensitization kinetics when compared with receptors containing other *α* subunits [97].

In the adult lumbar rat spinal cord, only *α*2, *α*3, *β*3, and *γ*2 mRNAs are expressed at significant levels, the *α*3, *β*3 and *γ*2 transcripts being present in many neurons throughout the Rexed laminae, whereas the *α*2 mRNA is restricted to motor neurons and adjacent cells [98]. A high expression level of the *α*1 and the *α*2 subunits is detected using immunohistochemistry in the adult rat oculomotor trochlear nuclei, the hypoglossal nucleus, and the dorsal nucleus of the vagus [99]. Interestingly, the motor trigeminal nucleus mainly expresses the *α*2 subunits, while *α*5 and *β*2/3 are poorly present in these CNS areas and the *δ* subunit is undetectable [99]. A recent immunohistochemical study, performed in human brainstem and cervical spinal cord, shows roughly similar results [100]. In this study, Waldvogel et al. did not analyze the expression of *α*4–*α*6 subunits and *δ* subunits, but they showed that *α*1, *α*2, *α*3, *β*2/3, and *γ*2 GABA_A_R subunits are largely detected in the brainstem motoneuron nuclei and in the lamina IX as well as, in less extend, in the lamina X of the cervical spinal cord [100]. However, their data, collected from human brain, differ from Fritschy's group results obtained from rat tissue. Indeed, Waldvogel et al. find a high expression of *α*1, *α*2, *α*3, and *β*2/3 subunits in the motor trigeminal nucleus, while the *γ*2 subunit was poorly expressed [100]. This could reflect differences in GABA_A_R subunit expression between species. However, because these two studies are based on a semi quantitative analysis of immunostaining, at a macroscopic level, discrepancies must be taken with caution. Effectively, it is well known that immunostaining, particularly for GABA_A_R subunits, can strongly vary depending on the fixation procedure [101, 102].

From a developmental point of view, little is known about changes in GABA_A_R subunit expression during spinal cord MNs development. In the rat cervical spinal cord, the *α*6 and *δ* subunits mRNAs are not detectable at all ages tested (from E12 to adult). During the ontogeny, as demonstrated for GABA [56, 57], subunits mRNA expression emerges along a ventrodorsal gradient. In fact, *α*2, *α*3, *α*5, *β*2, *β*3, *γ*2, and *γ*3 subunits emerge in presumptive MNs at E12–E13 and then can be detected in more dorsal regions [103]. A synchronized peak of *α*2, *α*3, *β*2, *β*3, *γ*2, and *γ*3 subunits mRNAs is detected at neonatal stages. In the adult rat cervical spinal cord, GABA_A_R *α*1, *α*4, *α*5, *β*1-2, *γ*1, and *γ*3 subunit mRNAs are found only in relatively few cells scattered in the gray matter, whereas mature MNs exhibit *α*2*β*3*γ*2 transcripts [103]. Thus, contrary to that observed for glycine receptors [104], there is no obvious switch in GABA subunit expression during prenatal and postnatal development of MNs. Interestingly, the *α*3 mRNA level observed at early developmental stage in the lateral motor column decreases around birth and was no longer detected in the adult [103]. In the hypoglossal nuclei, indirect proofs based on immunochemistry favor a switch from *α*1 to *α*2 subunits, during prenatal development [105]. As mentioned above, the *α*1 and *α*2 GABA_A_R subunits, together with the *γ*2 GABA_A_R subunit, are the main GABA_A_R subunits expressed in the hypoglossal nucleus of the adult rat [99]. Assuming that *γ*2 GABA_A_R clusters that do not colocalize with *α*1 GABA_A_R clusters reflect the presence of GABA_A_R containing *α*2 subunits, Muller and collaborators concluded for an increase in the proportion of GABA_A_R containing *α*2 GABA_A_R subunits [105]. However, this is in apparent contradiction to other studies showing that the *α*2 GABA_A_R subunits are expressed early in development and are progressively replaced by *α*1 GABA_A_R subunit in many brain areas [106]. A further quantitative immunohistochemical analysis of the developmental changes in the proportion of *α*2 and *α*1 GABA_A_R subunits in the hypoglossal nucleus is thus required in order to verify that developmental maturation processes of GABA_A_Rs can vary between CNS areas.

If it is now clearly demonstrated that GABA_A_R subunits may evolve during development and vary according to brain areas, few data are available concerning the cellular location of these subunits on a single MNs. Using immunocytochemistry and confocal microscopy, Lorenzo et al. compared the subcellular patterns of expression of the main GABA_A_R subunits (GABA_A_R  *α*1, *α*2, *α*3, and *α*5) in the somatic versus dendritic compartments of rat abducens MNs [107] and revealed a differential organization of GABA_A_R subunits. They found that MNs somata contain only GABA_A_R *α*1, while both GABA_A_R *α*1 and GABA_A_R *α*3 are detected on dendrites [107].

## 7. Maturation of the GABAergic System on Motoneuron in Normal and Pathological Conditions: Mixed GABA/glycine Synapses and Mismatch between Pre- and Postsynaptic Elements

**Figure 2:**
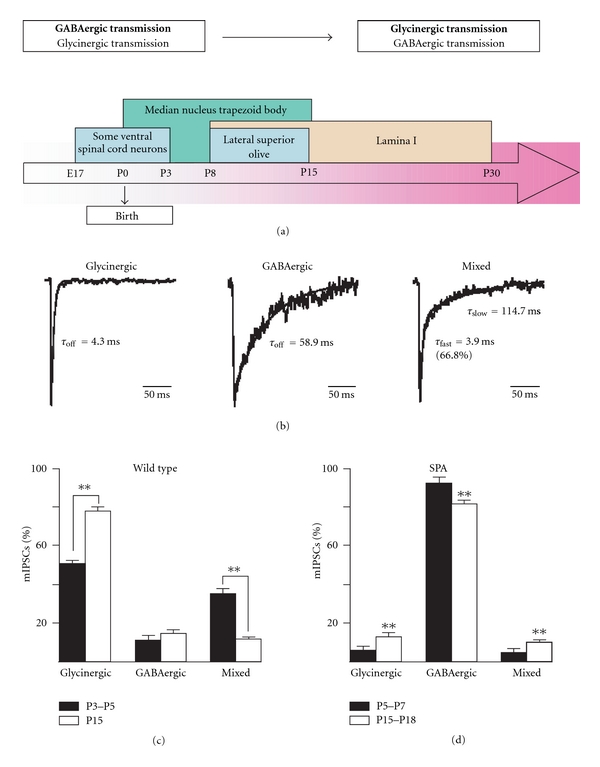
(a) Developmental changes in the proportions of GABAergic and glycinergic synaptic activity in various areas of the central nervous system. (b) Examples of individual glycinergic (left) GABAergic (middle) and mixed (right) miniature inhibitory postsynaptic currents (mIPSCs) recorded in a hypoglossal motoneuron at P15, in the presence of tetrodotoxin (a blocker of voltage-gated sodium channels). Note the slower decay phase of the GABAergic mIPSC compared to the glycinergic mIPSC. Decay phase of GABAergic and glycinergic events is better fitted with a single exponential function, while a double exponential function is required to fit the decay phase of mixed events. (c) Relative proportions of glycinergic, GABAergic and mixed mIPSCs at P3–P5 (black bars) and at P15 (white bars) in wild-type mice. (d) Relative proportions of glycinergic, GABAergic and mixed miniature postsynaptic events at P5–P7 (black bars) and at P15–P18 (white bars) in SPA mice. (Adapted from [118, 126]).

During the first 3 weeks of rodent postnatal development, inhibitory synaptic transmission changes in multiple ways that differ depending on brain areas. Electrophysiology and immunocytochemistry suggest that the respective contribution of the glycinergic and GABAergic transmission to the overall inhibitory message received by postsynaptic neurons may vary during the developmental period. For example, a developmental switch from a predominant GABAergic to main glycinergic neurotransmission occurs in the lumbar spinal cord [69] and in the lateral superior olive of young rodents [108, 109], while GABAergic neurotransmission dominates in developing collicular neurons [110] (Figure 2(a)). 

As first demonstrated in neonatal spinal MNs, glycine and GABA can be coreleased from the same presynaptic vesicle resulting in a mixed glycinergic/GABAergic synaptic event [111]. Mixed inhibitory synapses have also been functionally identified in MNs of the hypoglossal nucleus [112, 113], but mixed synapses are not particular to inhibitory input on MNs, because they are also described on spinal interneurons [114, 115]. If mixed inhibitory synapses appear to reflect an intermediate stage of maturation of glycinergic synapses, it must be noted that although the proportion of mixed synapses decreases during development in Renshaw cells and other spinal cord interneurons [116], mixed inhibitory synapses remain functional in the adult [114, 116]. This is also the case in abducens MNs during rat postnatal development: before birth, only GABAergic axon terminals develop, whereas mixed GABA/glycine axon terminals appear at birth, and their number increases during the first postnatal week [117].

Functional mixed inhibitory synapses have also been described in rat HMs [112, 113]. However, a complete morphofunctional study of the development of inhibitory synapse on the mouse HMs, between P3–P5 and P15, revealed that the developmental shift from glycinergic/GABAergic to pure glycinergic neurotransmission depends mainly on the maturation of the presynaptic elements, while postsynaptic GlyRs and GABA_A_Rs remain associated at the same postsynaptic density at all age tested. Effectively, although miniature inhibitory postsynaptic currents (mIPSCs) are mainly glycinergic and mixed glycinergic/GABAergic at P3–P5 and then predominantly glycinergic at P15 (Figures 2(b) and 2(c)), postsynaptic GlyRs and GABA_A_Rs remain associated at the same postsynaptic density at all age tested [118]. In addition, because many GABAergic synapses are unlikely to contain postsynaptic GABA_A_Rs yet, it was supposed that they represent newly formed ‘‘nonfunctional” GABAergic synaptic contacts, as previously observed in the cerebellum [119, 120]. It is, however, unclear whether such a discrepancy between the pre- and the postsynaptic element also occurs in other CNS area during development, but it must be noted that a similar maturation process of the inhibitory presynaptic terminals was also observed in neurons of the rat lateral superior olive [109]. Moreover, postsynaptic GABA_A_Rs facing presynaptic terminals that do not release GABA have also been reported in the spinal cord and brain neuropil in culture [121–125]. Such a mismatch between the pre- and the postsynaptic element of inhibitory synapses was also observed in the adult Renshaw cells of the rat spinal cord [114]. In that case, it was proposed that GABAergic presynaptic terminals could face postsynaptic GlyR clusters [114]. Altogether, these data suggest that the maturation of inhibitory synapses rather results from a differential regulation of the GlyT2 and GAD65 expression at the level of a single synaptic terminal but not from a redistribution of GlyRs and GABA_A_Rs at postsynaptic site.

Our data from the hypoglossal nucleus also suggest that pre- and postsynaptic elements mature independently [118]. However, a more recent study performed on spastic (SPA) mice, a model for hyperekplexia, argues against this hypothesis [126]. SPA mice display an insertion of an LINE-1 transposable element into the gene coding for the GlyR *β* subunit, which results in a truncated protein that impairs accumulation of GlyRs at postsynaptic sites and leads to a strong dysfunction of glycinergic synaptic transmission [127, 128]. In C57BL/6J strain, SPA mice which express a lower amount of GlyR *β* subunits die 2-3 weeks after birth [129], suggesting that GABAergic compensation does not necessarily take place. It was first hypothesized that the progressive postnatal developmental lost of GABAergic presynaptic terminals that normally occurs in wild-type mice due to a switch to glycinergic terminals [118] could explain the progressive impairment of inhibitory synaptic activity and thus the lethality of this mutation. But surprisingly, in opposition to our observations made in wild-type animal, the inhibitory synaptic activity is mainly GABAergic in SPA mice (Figure 2(d)): a developmental decrease in glycinergic presynaptic terminals occurs, while the density of GABAergic presynaptic terminals increases [126]. In addition, the proportion of inhibitory presynaptic terminals facing GABA_A_Rs significantly increases during postnatal development in HMs of SPA mice. It must, however, be noted that many GABAergic synaptic boutons face diffuse GABA_A_Rs staining, which contrasts to the situation observed in wild-type animal which most of the presynaptic terminals face aggregated GABA_A_Rs. It is, thus, likely that GABAergic synapses are less efficient in SPA mice than in wild type [126]. Also, because SPA mice cannot survive, these results indicate that GABAergic neurotransmission does not compensate for defects in GlyR postsynaptic aggregation in this hyperekplexia model. They also suggest, contrary to that previously hypothesized [118], that a crosstalk exists between postsynaptic and presynaptic elements, leading to the developmental regulation of the presynaptic terminal neurotransmitter content that could be related to a downregulation of GlyT2 expression and an up-regulation of GAD65 expression at inhibitory presynaptic terminals depending on the level of postsynaptic GlyR aggregation. 

Alteration of GABA_A_R and GlyR expression was also analyzed in MNs vulnerable and resistant to amyotrophic lateral sclerosis (ALS) [41]. Because a reduced level of expression of the GABA_A_R *α*1 subunit mRNA has been shown in neurons of the motor cortex of patients with ALS [130], Lorenzo et al. investigated, using a quantitative immunohistochemical study, the possibility that GABA_A_R and GlyR might be expressed differentially in ALS-vulnerable and ALS-resistant brainstem MNs in an ALS rat model [41]. Indeed, MNs controlling eye movements and bladder contraction are surprisingly unaffected (they are ALS-resistant) during terminal stages of ALS, while other MNs underlie an invariably fatal degeneration (they are ALS-vulnerable) [131]. Their main hypothesis was a reduction in GABA_A_R and GlyR expression in vulnerable MNs, which could account for an alteration of the inhibition and hence for an amplification of the glutamatergic synaptic activity onto these MNs, an excessive excitatory transmission being known to be detrimental. Interestingly, Lorenzo et al. showed a differential expression of GABA_A_R (and GlyR) in brainstem ALS-resistant oculomotor (III), trochlear (IV), abducens (VI) versus ALS-vulnerable MNs trigeminal (V), facial (VII), hypoglossal (XII) [41]. They demonstrated that GABA_A_R in ALS-vulnerable MNs mostly express *α*2 subunits while GABA_A_R in ALS-resistant MNs are *α*1 subunits enriched. They also showed that ALS-resistant MNs contain a larger proportion of extrasynaptic GABA_A_R clusters than ALS-vulnerable MNs. Because extrasynaptic GABA_A_R are activated by GABA spillover from synapses [132–134] and mediate a tonic inhibition that plays a crucial role in regulating neuronal excitability [135], the authors hypothesized that the presence of extrasynaptic GABA_A_R in ALS-resistant MNs could protect these neurons from excessive depolarization by abnormal glutamate release. Their data demonstrated that the rate of occurrence of extrasynaptic GABA_A_R clusters was approximately twice as high in ALS-resistant as in ALS-vulnerable MNs, but more experiments are necessary to determine to what extend this difference accounts for the vulnerability of MNs, as for example by manipulating extrasynaptic GABA_A_R expression in specific MNs. On the contrary, recent reports show that glycinergic innervation but not GABAergic innervation of spinal MNs is deficient in the ALS mouse model expressing the mutant form of human superoxide dismutase-1 with G93A substitution (SOD1^ G93A ^) [136, 137]. The authors examined, using whole-cell patch-clamp recordings, GlyR-mediated currents in cultured spinal MNs from this ALS mouse model. They found that glycine-evoked current density was significantly smaller in the SOD1 MNs compared to control. However, they did not find any change in GABAergic synaptic activity. This alteration in glycinergic synaptic activity is likely to be due to a lower GlyR*α*1 subunit mRNA expression in SOD1^G93A^ MNs [137]. These results suggest that a selective alteration in GlyR expression can partly account for an alteration of inhibitory synapse efficacy in MNs early in the disease process of ALS, with SOD1^G93A^ substitution at least. But these data obtained from GlyR expression in this ALS mouse model do not demonstrate, as data regarding GABA_A_R expression, that a reduction of receptor subunit expression can effectively account for MNs vulnerability in ALS. Again, more experiment is necessary to resolve this issue.

Finally, these results on GABA_A_R or GlyRs expression in ALS could be complementary rather than contradictory if one supposes that the expression of the different GlyR and GABA_A_R subunits can be region specific. For example, GABA_A_R *α*1 subunit is poorly expressed in the spinal cord compared to more central region [103], and it is important to note that glycinergic and GABAergic synapses control MNs development in a region-specific manner during programmed cell death as exemplified by data obtained in gephyrin-deficient mice that lack all postsynaptic GlyRs and some GABA_A_R clusters [138]. In these gephyrin-deficient mice, there is a reduced respiratory MN survival and decreased innervation of the diaphragm, whereas limb-innervating MNs show increased survival and increased innervation of their target muscles [138].

## 8. Concluding Remarks

If GABAergic interneurons constitute only 17%–20% of the neurons in the brain [139], their primordial role in the maintenance of a good balance in neuronal connections is obvious. GABA_A_R activation is likely to play an important role on spinal cord and brainstem MNs development as well as during pathological conditions, but it is unclear to what extend such a diversity leading to functionally different GABA_A_Rs is important for a proper development of functional locomotor networks and to what extend a defect in a subunit expression can impact neuronal survival during development and in pathological condition as in ALS. For example, it will be of interest to determine to what extend the expression of *α*2 GABA_A_Rs instead of *α*1 is important for neuronal development. This can be done using genetic tools as the knock in technique by substituting *α*2 expression by *α*1. Another unknown mechanism that must be determined is the communication pathway between GABAergic/glycinergic pre-synaptic neurons and post-synaptic receptors. Thus, it would be worthy to examine changes in the pre-synaptic GABAergic and/or glycinergic phenotype, during development or in pathological conditions, when a post-synaptic receptor type is missing or altered.
